# Modular design of metabolic network for robust production of *n*-butanol from galactose–glucose mixtures

**DOI:** 10.1186/s13068-015-0327-7

**Published:** 2015-09-04

**Authors:** Hyun Gyu Lim, Jae Hyung Lim, Gyoo Yeol Jung

**Affiliations:** Department of Chemical Engineering, Pohang University of Science and Technology, 77 Cheongam-Ro, Pohang, 37673 Gyeongbuk Korea; School of Interdisciplinary Bioscience and Bioengineering, Pohang University of Science and Technology, 77 Cheongam-Ro, Pohang, 37673 Gyeongbuk Korea

**Keywords:** Metabolic engineering, Synthetic biology, Galactose, Redox balance, Butanol

## Abstract

**Background:**

Refactoring microorganisms for efficient production of advanced biofuel such as *n*-butanol from a mixture of sugars in the cheap feedstock is a prerequisite to achieve economic feasibility in biorefinery. However, production of biofuel from inedible and cheap feedstock is highly challenging due to the slower utilization of biomass-driven sugars, arising from complex assimilation pathway, difficulties in amplification of biosynthetic pathways for heterologous metabolite, and redox imbalance caused by consuming intracellular reducing power to produce quite reduced biofuel. Even with these problems, the microorganisms should show robust production of biofuel to obtain industrial feasibility. Thus, refactoring microorganisms for efficient conversion is highly desirable in biofuel production.

**Results:**

In this study, we engineered robust *Escherichia coli* to accomplish high production of *n*-butanol from galactose–glucose mixtures via the design of modular pathway, an efficient and systematic way, to reconstruct the entire metabolic pathway with many target genes. Three modular pathways designed using the predictable genetic elements were assembled for efficient galactose utilization, *n*-butanol production, and redox re-balancing to robustly produce *n*-butanol from a sugar mixture of galactose and glucose. Specifically, the engineered strain showed dramatically increased *n*-butanol production (3.3-fold increased to 6.2 g/L after 48-h fermentation) compared to the parental strain (1.9 g/L) in galactose-supplemented medium. Moreover, fermentation with mixtures of galactose and glucose at various ratios from 2:1 to 1:2 confirmed that our engineered strain was able to robustly produce *n*-butanol regardless of sugar composition with simultaneous utilization of galactose and glucose.

**Conclusions:**

Collectively, modular pathway engineering of metabolic network can be an effective approach in strain development for optimal biofuel production with cost-effective fermentable sugars. To the best of our knowledge, this study demonstrated the first and highest *n*-butanol production from galactose in *E. coli*. Moreover, robust production of *n*-butanol with sugar mixtures with variable composition would facilitate the economic feasibility of the microbial process using a mixture of sugars from cheap biomass in the near future.

**Electronic supplementary material:**

The online version of this article (doi:10.1186/s13068-015-0327-7) contains supplementary material, which is available to authorized users.

## Background

Advances in metabolic engineering and synthetic biology have enhanced the ability to develop microbes for the efficient conversion of sugars to biofuels with high yield and productivity [1, 2]. *n*-Butanol especially has been spotlighted as the potential replacement for gasoline due to the similar energy properties to petroleum-based fuels [3]. Indeed, engineering efforts to improve the efficiency of *n*-butanol synthesis pathway showed significant improvement in microbial conversion. However, even with engineering successes in ‘drop in’ advanced biofuels [4, 5], an additional challenge lies in the production of desired chemicals from cost-effective feedstock to achieve industrial feasibility [6, 7].

One of the abundant sugars in cheap feedstock is galactose, mainly obtainable as a mixture of glucose from agar and cellulosic component of red seaweed, lactose of dairy waste, etc. [8, 9]. Galactose is an epimer of glucose and the only difference is the orientation of the hydroxyl group on the fourth carbon. However, the pathway for galactose assimilation in microorganisms is more complicated than glucose. This may lead to a more reduced rate for galactose utilization than that of glucose [10]. In addition, the carbon catabolite repression (CCR) for selective utilization of glucose hinders the simultaneous utilization of galactose and glucose and lowers the overall carbon flux toward central carbon metabolism [10, 11]. This is problematic because the cell factory should show robust performance with multiple carbohydrates regardless of carbon composition.

Recent studies in synthetic biology showed that modular pathway engineering could be successfully applied to develop and optimize artificial biological systems [12–14]. The redesigned metabolic genes with the predictable and quantitative regulatory elements at both the transcriptional and translational levels [15] can constitute individual modules. Following the assembly of these modules can rewire a whole metabolic network and finally construct the platform cell factory for the robust production of the various value-added chemicals from the various cost-effective carbon sources [16, 17]. In our previous works, we individually demonstrated robust modules for the efficient utilization of galactose [10] and production of *n*-butanol [18, 19] with the aid of synthetic constitutive promoters and tailored 5′-untranslated regions (UTR) in *E. coli*. In addition, we observed that the oxidized and reduced nicotinamide redox cofactors (NAD^+^ and NADH) acted as the important co-substrates in the each sugar utilizing and metabolite producing modules, and consequently, the overall efficiency of biofuel production was significantly influenced upon maintaining the ratio between NAD^+^ and NADH [18, 20]. Therefore, precisely regulated pathway of NADH supplementation is necessary to achieve the maximum production rate of *n*-butanol [18]. Collectively, the entire pathway from galactose to *n*-butanol can be further divided into three modules responsible for galactose-utilization, *n*-butanol formation, and the NADH supplementation.

In the present study, we carried out the assembly of the galactose utilization and *n*-butanol production modules designed to maximize the catalytic rates into the *E. coli* strain lack of other fermentative pathways. Subsequently, an NADH supplementation module, *fdh1* gene from *Saccharomyces cerevisiae* coding formate dehydrogenase, was introduced and its expression level was tuned for coordinating the galactose-utilizing and the *n*-butanol producing modules to accomplish maximization of the production rate of *n*-butanol from galactose. Finally, fermentation of mixtures of galactose and glucose at various composition ratios indicated that our cell factory could facilitate robust production of advanced biofuels regardless of sugar composition. The modular engineering of metabolic pathways, demonstrated in this work, will expedite the design and development of robust and efficient microbial cell factory for the production of various value-added chemicals from diverse cost-effective feedstock in nature.

## Results and discussion

### Design of galactose utilization and *n*-butanol production modules in *E. coli*

**Fig. 1:**
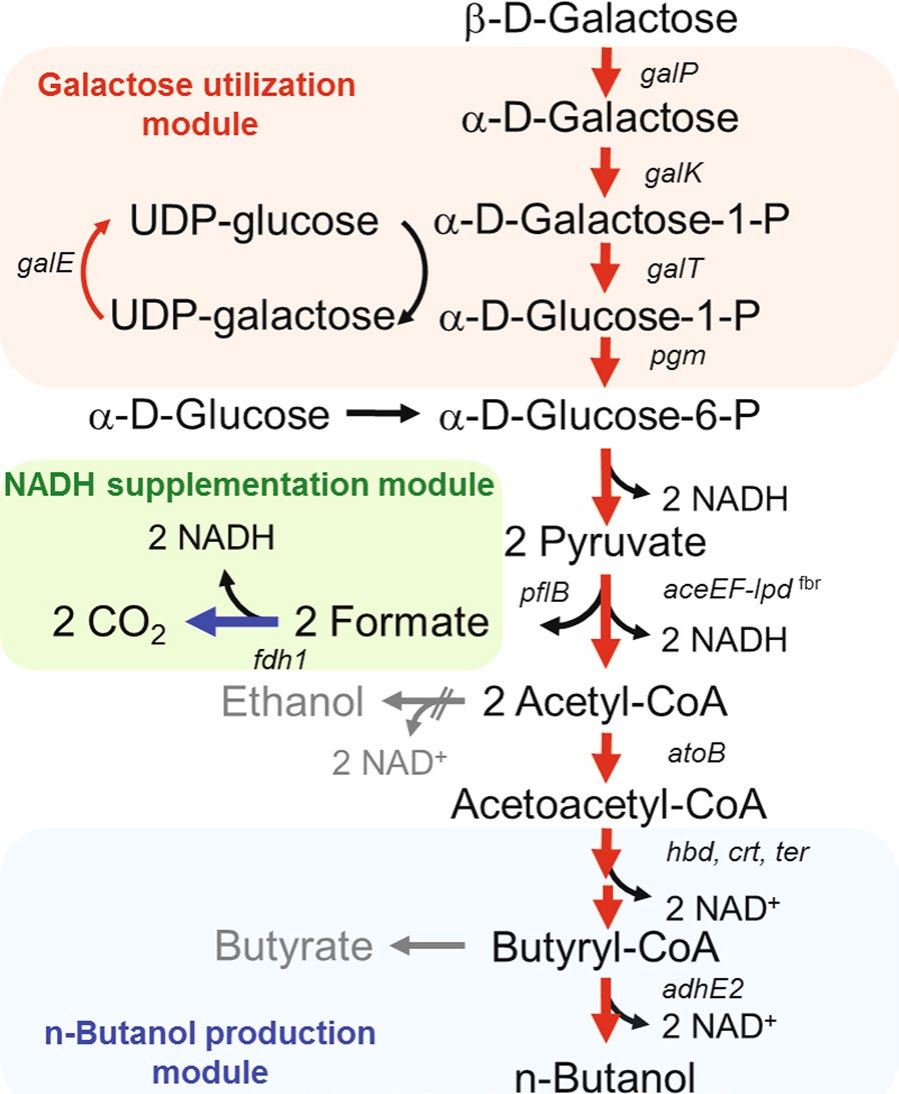
Schematic diagram of the assembled modules to convert galactose to *n*-butanol. In galactose utilization module, β-d-galactose is converted into α-d-glucose-6-phosphate through the Leloir pathway, which is a more complicated enzymatic pathway than that required for glucose. Acetoacetyl-CoA from α-d-glucose-6-phosphate is transformed into *n*-butanol by the *n*-butanol production module. Four moles of NADH generated from one mole of galactose are used in the *n*-butanol biosynthesis. In NADH supplementation module, additional NADH is produced by converting formate to carbon dioxide. The *red arrows* indicate the homologous and heterologous overexpression of genes. The *blue arrow* represents controllable expression of *fdh1* for optimal *n*-butanol production

The galactose metabolizing pathway, also known as the “Leloir pathway”, involves several enzymes that catalyze galactose catabolism in addition to glucose metabolism. As shown in Fig. 1, galactose is imported by galactose transporters (encoded by *galP*, and *mglBAC*) and is subsequently converted into glucose-1-phosphate through multiple enzymes encoded by the galactose operon (*galETKM*). Finally, phosphoglucomutase (encoded by *pgm*) converts glucose-1-phosphate to glucose-6-phosphate to enter glycolysis. In the presence of glucose, however, galactose metabolism is regulated by several factors, including a Gal repressor (GalR), Gal iso-repressor (GalS), and intracellular cyclic AMP, which induces CCR, enabling the preferential utilization of glucose. Although multiple enzymatic steps and the regulatory hierarchy in the pathway limit the rate of galactose utilization and the simultaneous fermentation of many carbohydrates [21], the reconstruction of galactose pathway on chromosome enhanced the galactose utilization rate and the simultaneous assimilation of galactose and glucose [10].

The assembly of both modules for synthetic galactose utilization and *n*-butanol production was rationally designed to enhance *n*-butanol production from increased galactose utilization. As the platform strain to introduce each module, we used previously reported *E. coli* strain JHL59 whose by-product pathways (acetate, lactate, ethanol, succinate) were blocked to amplify flux toward acetoacetyl-CoA [18]. For the galactose utilization module, the native galactose metabolism was engineered by deleting the Gal repressor (encoded by *galR*) and replacing the nascent regulatory elements on the “Leloir pathway”, including the enzymes encoded by *galP*, *galETKM*, and *pgm*, with predictable regulatory elements such as a synthetic constitutive strong promoter (BBa_J23100 from the Registry of Standard Biological Parts, http://partsregistry.org), and an optimized 5′-UTR designed by UTR Designer (http://sbi.postech.ac.kr/utr_designer) to amplify gene expression and avoid the host regulatory system [22]. Consequently, optimized galactose utilization module could be constructed (*E. coli* GAL_059).

In the case of the *n*-butanol production module, *n*-butanol, non-native metabolite in *E. coli*, is mainly produced by the introduction of four heterologous enzymes from *Clostridium acetobutylicum* and *Treponema denticola* with endogenous acetoacetyl-CoA as a starting material. Acetoacetyl-CoA (catalyzed by AtoB in *E. coli*) is firstly converted to 3-hydroxybutyryl-CoA by 3-hydroxybutyryl-CoA dehydrogenase (encoded by *hbd* from *C. acetobutylicum*). The crotonyl-CoA is then produced by crotonase (encoded by *crt* from *C. acetobutylicum*) from 3-hydroxybutyryl-CoA. Crotonyl-CoA is further modified to butyryl-CoA by *trans*-enoyl-CoA reductase (encoded by *ter* from *T. denticola*). Subsequently, *n*-butanol is finally produced with bifunctional aldehyde/alcohol dehydrogenase (encoded by *adhE2* from *C. acetobutylicum*) (Fig. 1). Accompanying deletion of by-product forming pathway, the overexpression of these heterologous enzymes with synthetic promoter and UTRs designed in the previous study [18] was utilized. Accordingly, the strain with both optimized modules could be obtained by transforming the pCDF-BuOH harboring maximized *n*-butanol production module [18] into *E. coli* GAL_059 (*E. coli* GAL_061).

### Enhanced *n*-butanol production of engineered strain with galactose

**Fig. 2:**
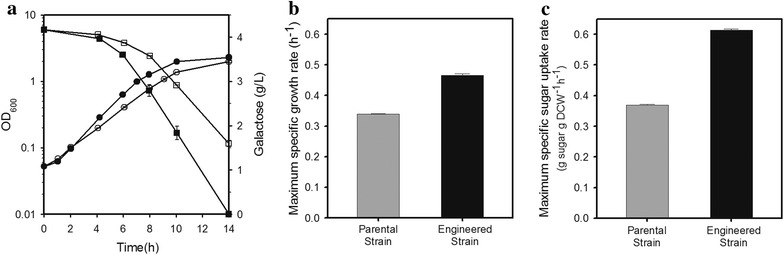
**a** Time-course of cellular growth and galactose consumption profiles of the parental (JHL59, *open symbol*) and engineered (GAL_059, *closed symbol*) strains in galactose supplemented minimal M9 medium for 14 h. The *left* *y*-axis represents OD_600_ in log scale and the *right* *y*-axis represents galactose concentration (g/L). The *x*-axis represents time in culture (h). Both the maximum specific growth rate (**b**) and the maximum specific sugar uptake rate (**c**) were higher in case of the engineered strain (GAL_059). The *error bars* indicate standard deviations of measurements from two independent cultures. Symbols: *circle* OD_600_; *rectangle* galactose

The *E. coli* GAL_059 strain was aerobically cultivated to evaluate its metabolic capacity when grown in M9 minimal medium containing galactose as a sole carbon source (Fig. 2a). As expected, the engineered strain showed a 37.2 % higher specific growth rate and a 66.4 % improvement in specific sugar uptake rate (Fig. 2b, c). The higher sugar uptake rate by the engineered strain resulted in the depletion of the galactose supplement in the medium within 14 h. In contrast, the parental strain JHL61 consumed about 60 % of the initial galactose.

**Fig. 3:**
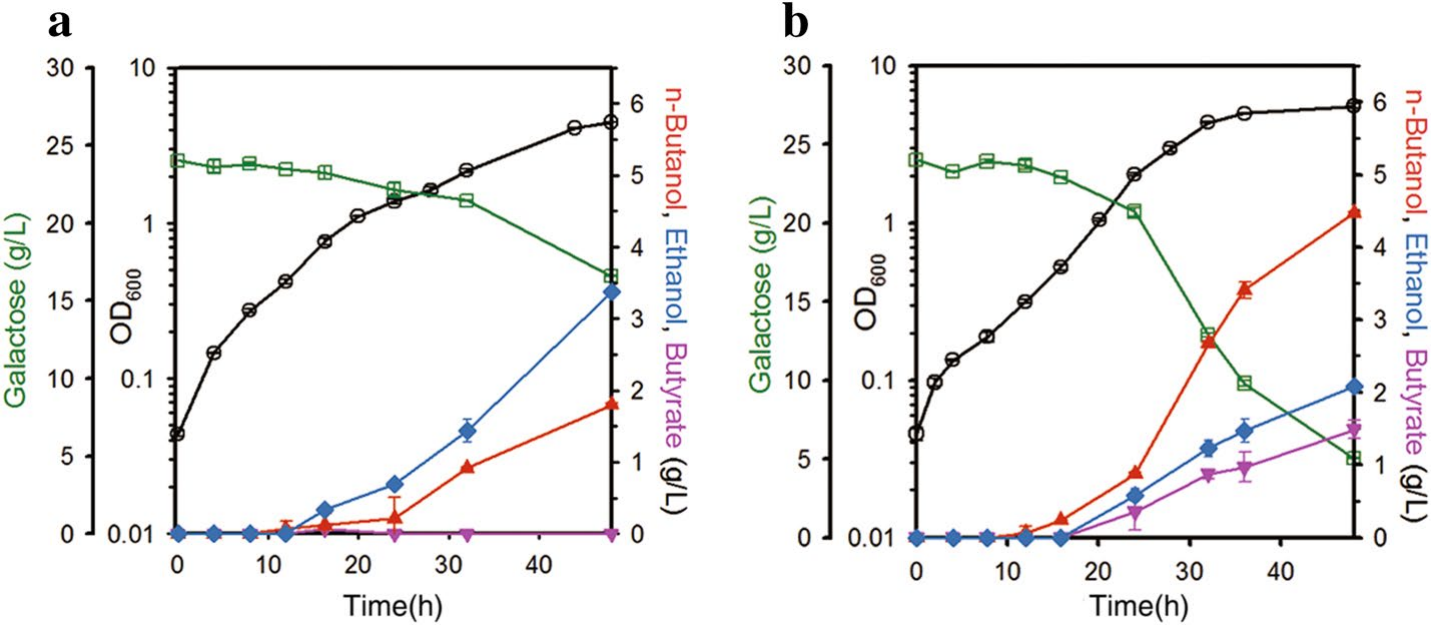
Time-course of fermentation profiles of the parental (JHL61, **a**) and engineered (GAL_061, **b**) strains for 48 h. Implementation of the galactose utilization pathway enhanced *n*-butanol production dramatically. The *left* *y*-offset and *right* *y*-axis represent galactose and metabolite (*n*-butanol, ethanol, and butyrate) concentrations (g/L), respectively. The *left* *y*-axis represents OD_600_. The *x*-axis represents time in culture (h). The *error bars* indicate standard deviations of measurements from two independent cultures. Symbols: *open circle* OD_600_; *open rectangle* galactose; *closed up triangle* *n*-butanol; *closed diamond* ethanol; *closed down triangle* butyrate

Furthermore, the *n*-butanol production capacity of our engineered strain GAL_061 was evaluated after anaerobic fermentation in galactose-supplemented medium for 48 h. After fermentation, this strain showed a dramatically increased final *n*-butanol titer of 4.5 g/L, about 2.5-fold higher than that produced by the parental strain. Moreover, the amount of galactose consumed was notably greater for this strain (19 g/L) than for the parental strain JHL61 (7.4 g/L), indicating that intracellular carbon flux was enhanced in the engineered strain (Fig. 3).

In addition, butyrate, which can be catalyzed by endogenous *tesB* (encoding acyl-CoA thioesterases) [19], was produced abnormally as a by-product in the engineered strain. This can be accounted for accumulation of butyryl-CoA (an intermediate of both *n*-butanol and butyrate) with increased carbon flux using galactose, probably due to insufficient activity of AdhE2 or the stoichiometric imbalance of reducing equivalent, NADH [23].

### Redox re-balancing by tuning the NADH supplementation module

Generally, increasing the availability of intracellular NADH is important to produce reduced metabolites, such as ethanol [24], succinate [25], and *n*-butanol [26, 27] and may be attainable by expressing yeast NAD^+^-dependent formate dehydrogenase (FDH1), an enzyme that converts formate to NADH and CO_2_ to supply additional NADH [25–27]. Our previous work elucidated that the optimal supply of NADH determined the efficiency of *n*-butanol production and was significantly different in galactose and glucose supplemented medium depending on the assimilation rate of sugars [18]. Moreover, the optimization of redox state was successfully accomplished through fine-tuning of FDH1 based on a predictive method of controlling translation using a biophysical thermodynamic model of the translation initiation process (UTR Designer) [22].

**Fig. 4:**
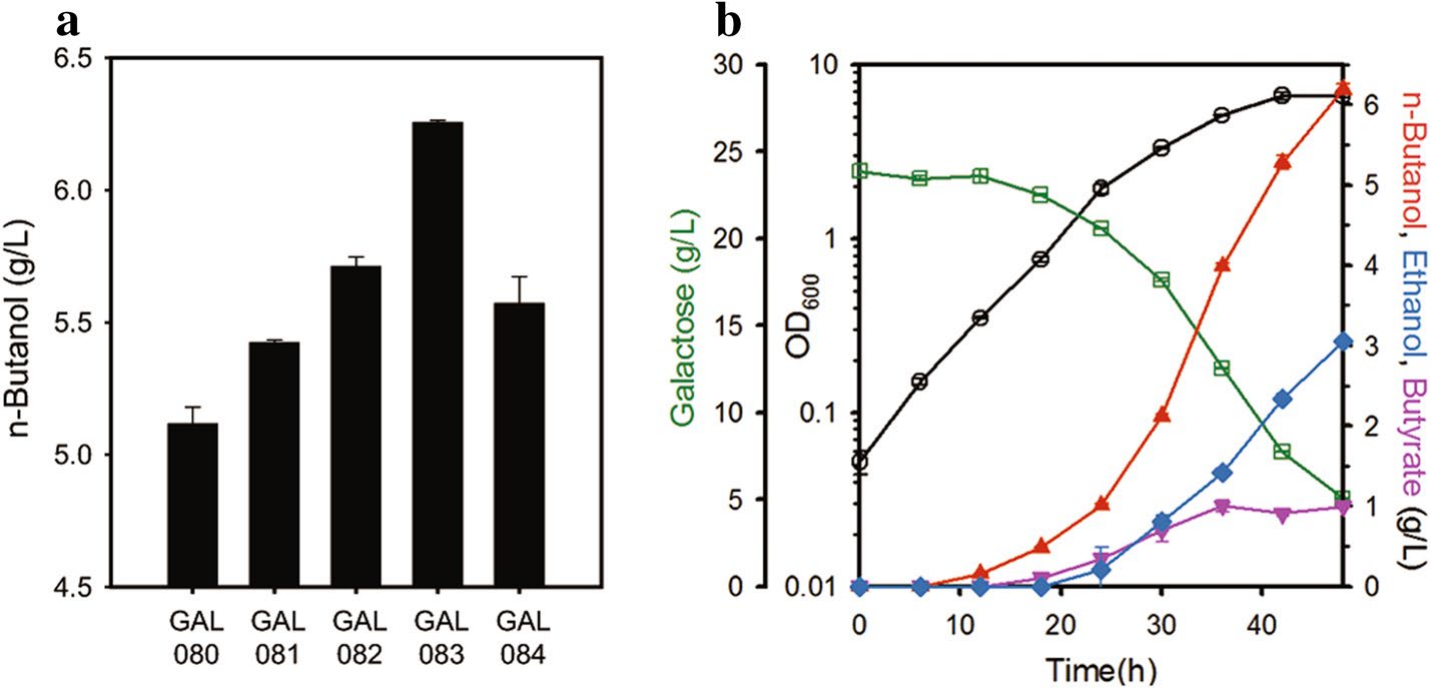
**a** Production of *n*-butanol by GAL_080 harboring pCOLADuet, GAL_081~084 harboring fdh1 variants after cultivation for 48 h in galactose-supplemented modified TB medium. **b** Time-course of fermentation profiles of the optimized strain (GAL_083), showing maximum *n*-butanol production. The pH was adjusted to around 7.2 at 6-h intervals. The *left* *y*-offset and *right* *y*-axis represent galactose and metabolite (*n*-butanol, ethanol, and butyrate) concentrations (g/L), respectively. The *left* *y*-axis represents OD_600_. The *x*-axis represents time in culture (h). The *error bars* indicate standard deviations of measurements from two independent cultures. Symbols: *open circle* OD_600_; *open rectangle* galactose; *closed up triangle* *n*-butanol; *closed diamond* ethanol; *closed down triangle* butyrate

In this manner, the commitment of expression cassettes for various levels of FDH1, termed as NADH supplementation module, was expected to effectively coordinate galactose-utilizing and *n*-butanol producing modules by optimization of intracellular redox state. To find the optimal redox state for *n*-butanol production equipped with galactose module, *fdh1* variants showing different levels of expression [18] were used to transform the GAL_061 strain, finally resulting in the GAL_080, 081, 082, 083, and 084 strains (Additional file 1: Table S1). After fermentation of the variants for 48 h, the relationship between *n*-butanol and *fdh1* expression level titer exhibited a concave curve (Fig. 4a). The carbon flux towards *n*-butanol was enhanced up to 22 % depending on the enzymatic activity of FDH1, indicating that the GAL_083 strain was redox balanced.

Optimizing the production of *n*-butanol increased the consumption of galactose and decreased the production of oxidized metabolites, such as pyruvate and butyrate, consistent with previous findings (Additional file 1: Table S3). Moreover, the optimal redox state in galactose-supplemented medium was markedly higher than that of the parental strain, indicating that increased carbon flux elevated the availability of NADH in our engineered strain [18]. Ultimately, the redox balanced strain GAL_083 produced 6.2 g/L of *n*-butanol after 48 h of fermentation (Fig. 4b). Collectively, the redox balancing module successfully optimized the catalytic amounts of reducing equivalents depending on carbon flux, resulting in maximum production of *n*-butanol from galactose.

### Simultaneous utilization of galactose and glucose for *n*-butanol production

Typically, biomass consists of a variety of polysaccharides with the composition depending on species, cultivation conditions, and methods of pretreatment [28–31]. Also, galactose is always obtainable as a sugar mixture of glucose from hydrolysate of seaweed, dairy waste [8, 9]. Therefore, the efficient conversion of cost-effective feedstock into valuable products requires the optimal, robust, and simultaneous consumption of galactose and glucose, regardless of their composition [11]. However, most microorganisms have evolved to utilize a preferred sugar in the presence of other sugars, enabling them to grow quickly under nutrient limited conditions [32]. The galactose metabolic pathway is repressed, both at the transcriptional and translational levels by glucose metabolism [33, 34].

We previously observed that deregulation and constitutive expression of transcripts whose 5′-UTR were designed for maximized expression allowed galactose to be utilized in the presence of glucose [10]. Accordingly, our engineered strain GAL_083 was expected to produce *n*-butanol through co-fermentation of galactose and glucose. To confirm this, GAL_083 and its parental strain were cultivated in simulated synthetic medium containing the same amounts of galactose and glucose that are generally present in seaweed and lactose [35]. Additionally, the ratios of these two sugars were varied from 2:1 to 1:2, reflecting the possible changes in the medium [29, 30, 36].

**Fig. 5:**
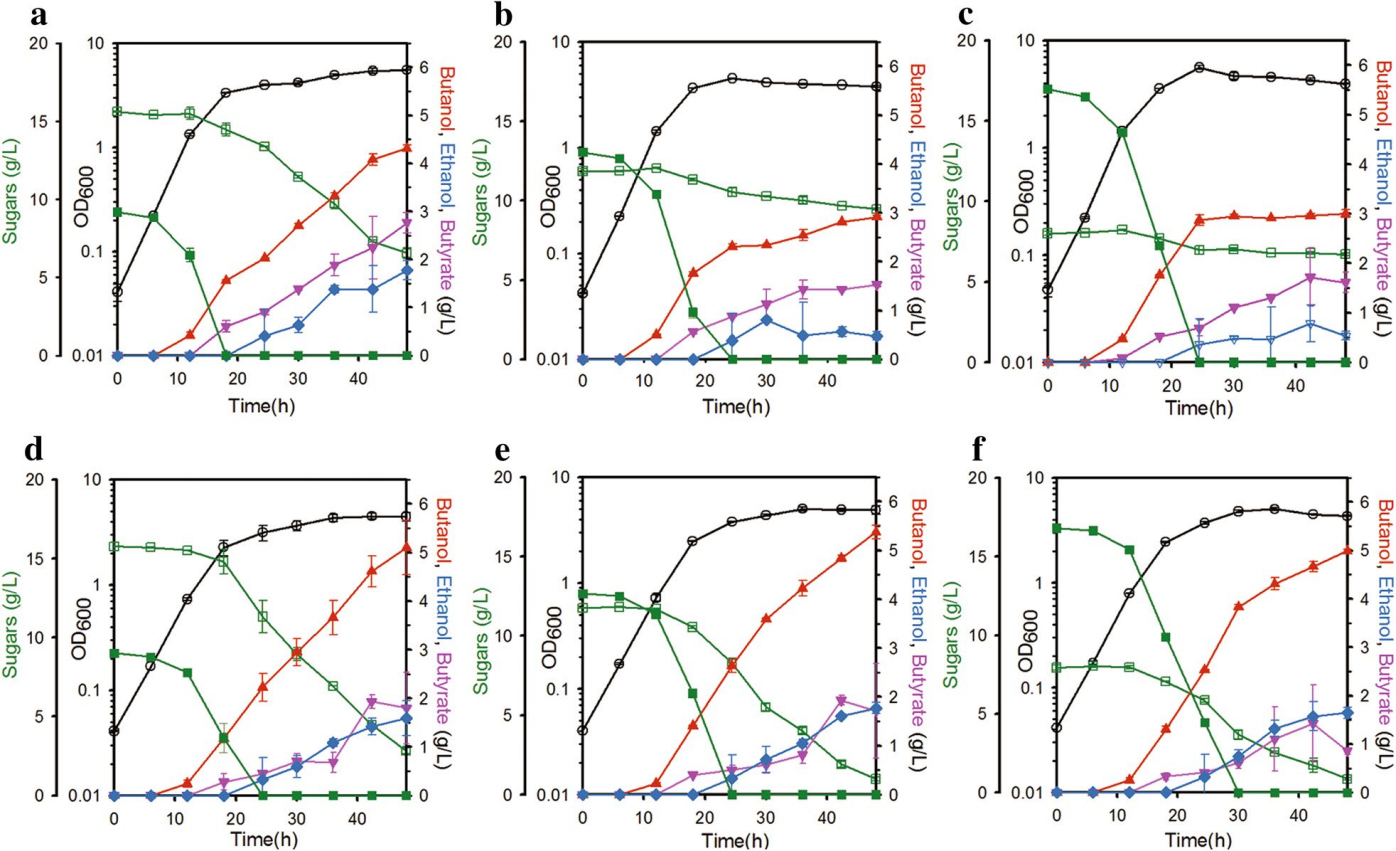
Time-course of fermentation profiles of the parental strain (JHL80, **a** through **c**) and redox optimized strain (GAL_083, **d** through **f**) in mixtures of galactose and glucose supplemented medium for 48 h. The pH was adjusted to around 7.2 at 6-h intervals. The ratio of galactose to glucose was varied from 2:1 (**a**, **d**), to 1:1 (**b**, **e**), to 1:2 (**c**, **f**). The GAL_083 strain showed the ability to utilize galactose and glucose simultaneously. The *left* *y*-offset and *right* *y*-axis represent sugar and metabolite (*n*-butanol, ethanol, and butyrate) concentrations (g/L), respectively. The *left* *y*-axis represents OD_600_. The *x*-axis represents time in culture (h). The *error bars* indicate standard deviations of measurements from two independent cultures. Symbols: *open circle* OD_600_; *open rectangle* galactose; *closed rectangle* glucose; *closed up triangle* *n*-butanol; *closed diamond* ethanol; *closed down triangle* butyrate

Under these conditions, the engineered strain utilized galactose and glucose simultaneously, while the parental strain showed no galactose consumption until glucose was depleted (Fig. 5). Moreover, *n*-butanol production by the engineered strain was consistently above 5 g/L regardless of the composition of the medium, while the yield of metabolites produced by the parental strain varied. Taken together, these results indicate that this strain would be promising as a platform strain for the optimal and robust production of valuable bio-chemicals from various galactose–glucose mixtures without regard to possible changes in the composition.

## Conclusion

In this study, synthetic galactose utilization and *n*-butanol production modules were assembled in *E. coli* to achieve efficient utilization of both galactose and galactose–glucose mixture. Furthermore, introduction of redox balancing module to optimize redox state depending on the enhanced carbon flux enabled the maximum production of *n*-butanol from galactose. The engineered GAL_083 strain showed a final titer of 6.2 g/L with a productivity of 0.13 g/L/h, both of which were much higher than those of the parental strain. Ultimately, the engineered strain could robustly co-ferment galactose and glucose regardless of sugar composition. To our best knowledge, this study demonstrated the first and highest *n*-butanol production from galactose in *E. coli*. Collectively, the modular pathway engineering could be broadly utilized to design microorganisms to convert a mixture of sugars from cheap biomass into advanced biofuels and other valuable chemicals.

## Methods

### Reagents, bacterial strains, and plasmids

The *E. coli* strains, plasmids, and primers used in this study are listed in Additional file 1: Table S1. Phusion DNA polymerase was purchased from New England Biolabs (Beverly, MA, USA). Oligonucleotides were synthesized by Genotech (Daejeon, Korea) and listed in Additional file 1: Table S2. Amplified DNA fragments were purified by GeneAll^R^ Expin^TM^ Gel SV kit (GeneAll Biotechnology, Seoul, Korea). Plasmids were prepared by AccuPrep^R^ Nano-Plus Plasmid Mini Extraction Kit (Bioneer, Daejeon, Korea). The reagents for cell culture were purchased from BD Biosciences (Sparks, MD, USA). Other reagents were obtained from Sigma (St. Louis, MO, USA).

All chromosomal manipulations were performed using the Red recombination with pKD46 and pCP20 [37, 38]. The synthetic galactose utilization pathway was constructed by replacing the nascent pathway of the JHL59 strain with synthetic regulatory elements, as described [10, 39]. To construct the GAL_059 strain, *galR* was deleted by insertion of a *FRT*-*Kan*^*R*^-*FRT* fragment amplified using D-galR-F/D-galR-R primers. The native *galETKM* operon was deleted by insertion of a *FRT*-*Kan*^*R*^-*FRT* fragment amplified using D-galETKM-F/D-galETKM-R primers and the resultant synthetic operon was introduced by PCR amplifying of pACYC_galO as a template with the O-galETKM-F/O-galETKM-R primers. To overexpress *galP* and *pgm*, *FRT*-*Kan*^*R*^-*FRT* fragments were amplified by O-galP-F/O-galP-R1/O-galP-R2 and O-pgm-F/O-pgm-R, respectively. The GAL_059 strain, which had deleted competing pathways (*adhE*, *ldhA*, *paaFGH*, *frdABCD*, *pta*) and overexpressed the producing pathway (*atoB*, *lpd*-G1060A, *aceEF*), was transformed with pCDF-BuOH containing heterologous *n*-butanol producing enzymes (*crt*, *hbd*, *ter*, *adhE2*) to yield the GAL_061 strain. GAL_080~084 strains harboring *fdh1* variants for redox optimization were prepared by electrical transformation of the GAL_061 strain with the plasmids designated in Additional file 1: Table S1.

### Media and growth conditions

To evaluate galactose assimilation rates, *E. coli* strains were cultivated in M9 minimal medium supplemented with galactose (4 g/L), M9 salt solutions, 5 mM MgSO_4_, and 0.1 mM CaCl_2_. The cells were subsequently cultivated in modified Terrific Broth, consisting of 12 g tryptone, 24 g yeast extract, 2.31 g KH_2_PO_4_, and 12.54 g K_2_HPO_4_ per liter, supplemented with a 25 g/L carbon source for *n*-butanol production, but without added glycerol. Multiple plasmids were maintained by including 25 μg/mL of streptomycin and 15 μg/mL of kanamycin in the media; single plasmid was maintained in media containing 50 μg/mL alone. The bacteria were cultured anaerobically in rubber-sealed, 60 mL serum bottles and an anaerobic chamber (Coy Laboratories, Ann Arbor, MI, USA) containing 97.5 % nitrogen and 2.5 % hydrogen gas. Oxygen was continuously removed by reaction with hydrogen in the presence of a palladium catalyst. The cells were cultured at 37 °C with shaking (250 rpm), and bacterial density was determined by measuring optical density at 600 nm (OD_600_) using a UV-1700 spectrophotometer (Shimadzu, Kyoto, Japan).

Seed cultures were aerobically prepared by inoculating colonies from an LB plate into 3 mL of galactose-containing M9 minimal medium. This initial seed culture was again inoculated under the same conditions with an OD_600_ of 0.05. When the OD_600_ of second-round seed cultures reached 1.0, the cultures were individually inoculated into M9 or modified TB. Unless otherwise stated, all bacteria were cultivated anaerobically. The culture pH was adjusted to around 7.2 using 10 M NaOH at every 6 h. Samples were frozen at −80 °C until analysis. All cell culture experiments were performed in duplicate.

### Analytical methods

The concentrations of sugars and metabolites were determined by UltiMate^TM^ 3000 analytical HPLC systems (Dionex, Sunnyvale, CA, USA) using an HPX-87H column (Bio-Rad Laboratories, Richmond, CA, USA) with a flow rate of 0.6 mL/min at 14 °C using 5 mM H_2_SO_4_ as the mobile phase. Signals were monitored with a Shodex RI-101 refractive index detector (Shodex, Klokkerfaldet, Denmark) and a UV–Vis diode array detector at 210 nm.

## Additional file

Additional file 1: Supplementary tables. **Table S1.** Strains and plasmids used in this study. **Table S2.** Primers used in this study. **Table S3.** Carbon balance table for the fermentation of engineered strains. Supplementary reference.

## Abbreviations

CCR: carbon catabolite repression
UTR: untranslated region
NAD^+^: oxidized nicotinamide adenine dinucleotide
NADH: reduced nicotinamide adenine dinucleotide
AMP: adenosine monophosphate
CoA: coenzyme A
Kan^R^: kanamycin resistance gene
OD: optical density
PCR: polymerase chain reaction
