# Supplementary material for: Modular design of metabolic network for robust production of n-butanol from galactose–glucose mixtures
Source: Biotechnol Biofuels. 2015 Sep 4;8:137. doi: 10.1186/s13068-015-0327-7 (PMC4559943; doi:10.1186/s13068-015-0327-7)
Supplement: Additional file 1: — Supplementary tables. Table S1. Strains and plasmids used in this study. Table S2. Primers used in this study. Table S3. Carbon balance table for the fermentation of engineered strains. Supplementary reference. [file 13068_2015_327_MOESM1_ESM.doc]

**Supplementary Tables**

**Table S1.** Strains and plasmids used in this study.

| **Name** | | **Relevant characteristics** | | **Source** |
| --- | --- | --- | --- | --- |
| **Strains** |  | |  | |
| Mach1-T1^R^ | F^-^ φ80(*lac*Z)ΔM15 Δ*lac*X74 *hsd*R(r_K_^-^m_K_^+^) Δ*rec*A1398 *end*A1 *ton*A | | Invitrogen | |
| W3110 | F^-^ λ^-^ rph-1 IN(rrnD, rrnE)1 | | ATCC 27325 | |
| JHL59 | W3110 Δ*ato*DA Δ*adhE* Δ*ldh*A Δ*paa*FGH  Δ*frd*ABCD Δ*pta* P*_ato_*_B_::BBa_J23100  P*_lpd_*::BBa_J23100 *lpd*(G1060A)  P*_aceEF_*::BBa_J23100 | | [1] | |
| JHL61 | JHL59/pCDF-BuOH | | [1] | |
| JHL80 | JHL61/pCOLADuet | | [1] | |
| GAL_059 | JHL59/Δ*galR* P_galP_-UTR_galP_:: PBBa_J23100-SynUTR_galP_ *galETKM*:: PBBa_J23100-SynUTR_galE_-*galE*-PBBa_J23100-SynUTR_galT_-*galT***-**PBBa_J23100-SynUTR_galK_-*galK***-**PBBa_J23100-SynUTR_galM_-*galM* P_pgm_-UTR_pgm_:: PBBa_J23100-SynUTR_pgm_ | | This study | |
| GAL_061 | GAL_059/pCDF-BuOH | | This study | |
| GAL_080 | GAL_061/pCOLADuet | | This study | |
| GAL_081 | GAL_061/pCOLA-F1 | | This study | |
| GAL_082 | GAL_061/pCOLA-F2 | | This study | |
| GAL_083 | GAL_061/pCOLA-F4 | | This study | |
| GAL_084 | GAL_061/pCOLA-F5 | | This study | |
|  |  | |  | |
| **Plasmids** |  | |  | |
| pKD46 | Red recombinase expression vector; Amp^R^ | | [2] | |
| pCP20 | FLP expression vector; Amp^R^ | | [2] | |
| pCOLADuet | Expression vector, ColA ori, Km^R^ | | Novagen | |
| pACYCgalO | p15A ori, Cm^R^,FRT-Kan^R^-FRT-PBBa_J23100-SynUTR_galE_-*galE*-PBBa_J23100-SynUTR_galT_-*galT***-**PBBa_J23100-SynUTR_galK_-*galK***-**PBBa_J23100-SynUTR_galM_-*galM* | | [3] | |
| pCDF-BuOH | cloDF13 ori, Sm^R^, P_J23100_::*crt*- P_J23100_::*hbd*- P_J23100_::*ter*-P_J23100_::*adhE2* | | [1] | |
| pCOLA-F1 | ColA ori, Km^R^, P_J23100_::F1_UTR_- *fdh1*_SC_ | | [1] | |
| pCOLA-F2 | ColA ori, Km^R^, P_J23100_::F2_UTR_- *fdh1*_SC_ | | [1] | |
| pCOLA-F4 | ColA ori, Km^R^, P_J23100_::F4_UTR_- *fdh1*_SC_ | | [1] | |
| pCOLA-F5 | ColA ori, Km^R^, P_J23100_::F5_UTR_- *fdh1*_SC_ | | [1] | |
|  |  | |  | |

**Table S2.** Primers used in this study.

| **Name^a^** | **Sequence (5’-3’)^b^** |
| --- | --- |
| D-galR-F | ctctgggatcaccactttagcaacctgaagccaaacgccaccagcggtcgcatgaccggcgcgatgc |
| D-galR-R | gctggaattgctttaactgcggttagtcgctggttgcatgatgacttgccgctcagcggatctcatgcgc |
| D-galETKM-F | cttagcaccctctccggccaacggttcgacgcatgcaggcatgaaaccgcccgcatgaccgcgcgatgc |
| D-galETKM-R | ctggtgatttgaacaatatgagataaagccctcatgacgagggcgtaacacgcgacgacaggcacatgcg |
| O-pgm-F | cgatgcaattcccggcggaattgattgagaaggtttgcggaactatctaaacacatttaataaaaaaagggcggtcgcaagatcgcccttttttgcatgaccggcgcgatgc |
| O-pgm-R | taatattgcgccgtcagttgggcgacgttaatcaaatcactctgttgtgctggttgacctgctctattatgaattgccattagtatctcctcctttacgactcctgctagcactgtacctaggactgagctagccgtcaagctcagcggatctcatgcgc |
| O-galP-F | gcccgcacaataacatcattcttcctgatcacgtttcaccgcagattatcccgcatgaccgcgcgatgc |
| O-galP-R1 | cctccttattaatatgaaatgctagcactgtacctaggactgagctagccgtcaacgcgacgacaggcacatgcg |
| O-galP-R2 | gcagacgaaaaacgtcattgccttgtttgaccgcccctgttttttagcgtcaggcatttattcctccttattaatatgaaatgctagcac |
| O-galETKM-F | cttagcaccctctccggcc |
| O-galETKM-R | ctggtgatttgaacaatatgagataaagcc |
| C-galR-F | gcacgacgactcttcgccag |
| C-galR-R | gggcgatgtctttacccagcagg |
| C-pgm-F | gctttcggatgaatacgcagagc |
| C-pgm-R | cggacagggcgtgagtatc |
| C-galP-F | ggtcgtgaacatttcccgtg |
| C-galP-R | gaacatcatggagcttacgaccc |
| C-galETKM-F | cgctgaatcgccagcttatccg |
| C-galETKM-R | ccagaaccagcttagttacagcc |

^a^Names beginning with “C” indicate primers used to check homologous recombination.

^b^Sequences were originally designed in our previous work [3]. Underlined letters indicate homologous sequences for recombination.

**Table S3.** Carbon balance table for the fermentation of engineered strains.

| **Strains** | **Biomass^a^** | **Galactose**  **Consumed (g/L)** | **Metabolites (g/L)^b^** | | | | **Carbon recovery^c^  (%)** |
| --- | --- | --- | --- | --- | --- | --- | --- |
|  |  |  | **n-Butanol** | **Butyrate** | **Ethanol** | **Pyruvate** |  |
| **JHL61** | **4.47±0.00** | **7.44.±0.02** | **1.80±0.02**  **(0.24, 60%)** | **ND** | **3.37±0.03** | **ND** | **116** |
| **GAL_061** | **5.53±0.21** | **19.00±0.02** | **4.47±0.02**  **(0.24, 59%)** | **1.48±0.13** | **2.09±0.07** | **3.24±0.01** | **89** |
| **GAL_080** | **5.34±0.15** | **16.85±0.13** | **5.12±0.06**  **(0.30, 76%)** | **1.58±0.09** | **1.52±0.12** | **3.97±0.06** | **107** |
| **GAL_081** | **5.45±0.18** | **18.32±0.01** | **5.42±0.01**  **(0.30, 74%)** | **1.64±0.00** | **1.67±0.08** | **3.23±0.06** | **99** |
| **GAL_082** | **5.94±0.04** | **18.21±0.03** | **5.71±0.04**  **(0.31, 78%)** | **1.49±0.08** | **1.51±0.02** | **2.20±0.01** | **95** |
| **GAL_083** | **6.32±0.28** | **18.92±0.47** | **6.25±0.01**  **(0.33, 83%** | **1.01±0.04** | **2.61±0.01** | **ND** | **89** |
| **GAL_084** | **5.20±0.07** | **17.97±0.27** | **5.57±0.10**  **(0.31, 78%)** | **1.49±0.06** | **3.64±0.07** | **ND** | **97** |

Data obtained after cultivating for 48 hours in galactose-supplemented TB medium. It should be noted that other components present in the TB medium can slightly affect to the titer of n-butanol and also other metabolites. The error bars indicate standard deviations of measurements from two independent cultures.

ND : Not detected

^a^Biomass represented as value of OD600 and one OD600 unit corresponds to 0.25 g dry cell weight (DCW)/ L.

^b^Numbers in parentheses indicate the yield (g of n-butanol/g of galactose) and % of theoretical maximum yield (0.41 g/g) .

^c^A C-mol of biomass calculated with a molecular formula of CH1.77O0.49N0.24P0.017 (MW=25.48) and average ash content of 5.5% [4]. An evolving CO2 during fermentation was not considered in this calculation.

**Supplementary References**

1. Lim JH, Seo SW, Kim SY, Jung GY. **Model-driven rebalancing of the intracellular redox state for optimization of a heterologous n-butanol pathway in Escherichia coli**. Metab Eng. 2013, **20**:56-62.

2. Datsenko KA, Wanner BL. **One-step inactivation of chromosomal genes in Escherichia coli K-12 using PCR products**. Proc Natl Acad Sci U S A. 2000, **97**(12):6640-5.

3. Lim HG, Seo SW, Jung GY. **Engineered Escherichia coli for simultaneous utilization of galactose and glucose**. Bioresour Technol. 2013, **135**:564-7.

4. Stephanopoulos G, Aristidou AA, Nielsen J. **Metabolic engineering: principles and methodologies**. San Diego, California, p.120: Academic Press; 1998.
